# Dual Characters of GH-IGF1 Signaling Pathways in Radiotherapy and Post-radiotherapy Repair of Cancers

**DOI:** 10.3389/fcell.2021.671247

**Published:** 2021-06-09

**Authors:** Yunyun Cheng, Wanqiao Li, Ruirui Gui, Chunli Wang, Jie Song, Zhaoguo Wang, Xue Wang, Yannan Shen, Zhicheng Wang, Linlin Hao

**Affiliations:** ^1^NHC Key Laboratory of Radiobiology, School of Public Health, Jilin University, Changchun, China; ^2^College of Animal Science, Jilin University, Changchun, China; ^3^The First Hospital of Jilin University, Changchun, China

**Keywords:** GH-IGF1 signaling pathway, cancers, radiotherapy, radio-resistance, DNA damage repair

## Abstract

Radiotherapy remains one of the most important cancer treatment modalities. In the course of radiotherapy for tumor treatment, the incidental irradiation of adjacent tissues could not be completely avoided. DNA damage is one of the main factors of cell death caused by ionizing radiation, including single-strand (SSBs) and double-strand breaks (DSBs). The growth hormone-Insulin-like growth factor 1 (GH-IGF1) axis plays numerous roles in various systems by promoting cell proliferation and inhibiting apoptosis, supporting its effects in inducing the development of multiple cancers. Meanwhile, the GH-IGF1 signaling involved in DNA damage response (DDR) and DNA damage repair determines the radio-resistance of cancer cells subjected to radiotherapy and repair of adjacent tissues damaged by radiotherapy. In the present review, we firstly summarized the studies on GH-IGF1 signaling in the development of cancers. Then we discussed the adverse effect of GH-IGF1 signaling in radiotherapy to cancer cells and the favorable impact of GH-IGF1 signaling on radiation damage repair to adjacent tissues after irradiation. This review further summarized recent advances on research into the molecular mechanism of GH-IGF1 signaling pathway in these effects, expecting to specify the dual characters of GH-IGF1 signaling pathways in radiotherapy and post-radiotherapy repair of cancers, subsequently providing theoretical basis of their roles in increasing radiation sensitivity during cancer radiotherapy and repairing damage after radiotherapy.

## Introduction

The growth hormone-insulin-like growth factors (GH-IGFs) growth axis plays widely roles in varieties of systems, including nervous system, reproductive system, skeletal system, muscular system, and immune system (Lu et al., 2019). As a growth axis, it is of great significance in the regulation of the growth and development of the body by promoting cell proliferation and inhibiting apoptosis. Based on these effects, many studies have reported its role in cancers, such as prostate, lung, colorectal, ovarian cancer, breast cancer, thyroid cancer, and liver cancer (Gunnell et al., 2001; Lango Allen et al., 2010; Chhabra et al., 2011; Wu and Gao, 2018), indicating that the effects of GH and IGF1 on cell proliferation can not only maintain the growth and development of normal organism, but also promote the process of cancer cells.

At present, radiotherapy is still a critical treatment for cancer therapy, including head, skin, cervical, laryngeal, nasopharyngeal tumors, prostate, and breast cancers (Cavalieri et al., 2018; Mudgal et al., 2019). Radiotherapy inhibits tumor growth by generally inducing tumor cell apoptosis, senescence, autophagy, necrosis, or mitotic catastrophe through inflicting extensive DNA damages (Baskar et al., 2012; Chen and Kuo, 2017). However, the roles of GH and IGF1 in promoting cell proliferation and inhibiting cell apoptosis simultaneously increase the difficulty in cancer radiotherapy due to their contributions to radio-resistance. Simply, GH and IGF1 regulate the process by promoting cell proliferation through janus kinase 2/signal transducer and activator of transcription 5 (JAK2/STAT5) signaling pathway or phosphatidylinositol 3-kinase/protein kinase B (PI3K/Akt) and mitogen-activated protein kinase/extracellular regulated MAP kinase 1/2 (MAPK/ERK1/2) pathways, inducing homologous recombination (HR) or non-homologous end joining (NHEJ) to repair DNA damage and decreasing pro-apoptotic molecules (Bax, caspase3) (Valenciano et al., 2012; Basu and Kopchick, 2019). Thus, radio-resistance caused by GH and IGF1 is not conducive to radiotherapy for cancer.

Besides, radiotherapy inevitably causes collateral damage to adjacent tissues at the same time. The most common damage of radiation to cells is based on its damage to DNA and subsequent genomic instability. To limit genomic instability, the cells have a series of repair proteins that engage the appropriate DNA repair pathways, and then produce some damage repair effects. Hormones like GH and IGF1 activate the related pathways by binding to their cognate receptors can decrease the sensitivity to irradiation and increase the expression levels of ataxia telangiectasia mutated (ATM), γH2AX, p53BP-1, catalytic subunits of the DNA-dependent protein kinase (DNA-PKcs) and PARP-1 that related to the DNA repair function (Chesnokova and Melmed, 2020), therefore achieving the aim of DNA damage repair.

In this review, we summarized the studies on GH-IGF1 signaling in the development of cancer, the resistance effects of radiotherapy in the treatment of cancer, and the repair of adjacent tissues after radiotherapy. Then we clarified the mechanisms of GH-IGF1 signaling in radio-resistance and tissue damage repair by its roles in promoting cell proliferation and attenuated apoptosis, as well as the critical DNA damage repair.

## Somatotrophic System: A Brief Overview of Structure and Physiological Function

**FIGURE 1:**
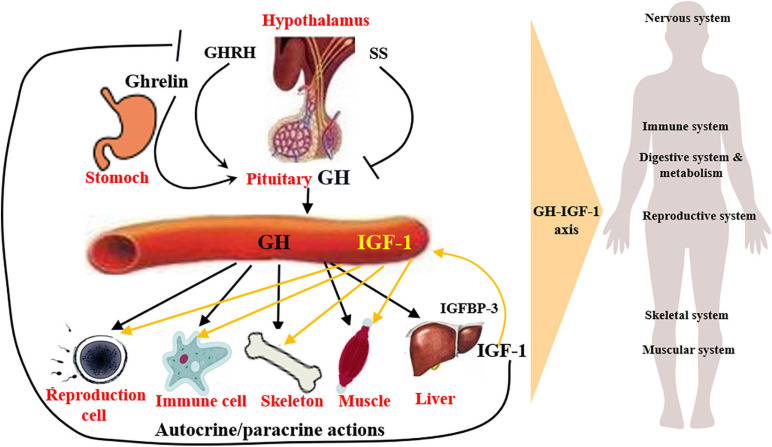
Structure and function of somatotrophic system. Somatotrophic system begins with hypothalamus, through the pituitary to the liver, ends in the target organs, impacting numerous physiological systems, such as nervous system, skeletal system, muscular system, reproductive system, and immune system, with wide-ranging effects. It involves circulating GH and IGF1, and the local production of GH and IGF1, which are the main determinant of postnatal body growth.

Somatotrophic system is the most important genetic and the terminal regulating factor affecting body growth and development (Ernst and Rodan, 1990; Sjögren et al., 1999), which impacts numerous systems with wide-ranging effects, like nervous system, reproductive system, muscular system, skeletal system, and immune system (Lu et al., 2019). The somatotrophic system involves circulating GH and IGF1, and the local production (autocrine or paracrine) of GH and IGF1 in several tissues (Figure 1). Autocrine GH for instance, is thought to be more oncogenic than pituitary GH, and the autocrine/paracrine IGF1 is the main determinant of postnatal body growth, including muscle and bone tissue development, confirming the conclusion that pituitary GH and circulating IGF1 are critical for body size and affect numerous body functions, for these body functions, local GH and IGF1 may be more relevant. (Dehkhoda et al., 2018).

Growth hormone is mainly produced by the anterior pituitary gland, secreted in a pulsatile manner and under direct control of hypothalamic neuronal projections (Pombo et al., 2001), whose secretion is mainly regulated positively by growth hormone releasing hormone (GHRH), ghrelin (Dimaraki and Jaffe, 2006; Khatib et al., 2014), and negatively by somatostatin (SS) (Lu et al., 2019). Following release, the action of GH is achieved through binding with GH receptor (GHR), which is widely expressed in variety of tissues (Møller and Jørgensen, 2009) and their interaction mainly results in the activation of JAK2/STAT5, MAPK-ERK1/2, PI3K-Akt, as well as PLC/PKC/Ca^2+^ signaling pathways (Zhu et al., 2001; Bocharov et al., 2018; Basu and Kulkarni, 2019; Chhabra et al., 2019) and the synthesis of IGF1 is mainly in liver (Guevara-Aguirre et al., 2018; Zhang et al., 2019; Firmenich et al., 2020). In the GH signaling, GH binding proteins are produced at the cell surface and play complex roles, including modulate the half-life of plasma GH and the binding of GH to GHR (Cawley et al., 2013).

In most cases, the growth-promoting effects of GH depend on IGF1. In general, GH promotes *IGF1* gene transcription and synthesis in the liver, thus regulating the circulating levels of IGF1 (Guevara-Aguirre et al., 2018; Firmenich et al., 2020). Hypophysectomized rats show a rapid increase in serum IGF1 after recombinant human GH (rhGH) administration, and a 75% reduction of circulating IGF1 levels due to the liver IGF1-deficient in mice leads to a fourfold increase in GH secretion (Yakar et al., 2004). While treatment with rhGH in normal mice increases body weight, lean body mass, and liver weight but does not increase hepatic expression and release of IGF1, suggesting IGF1 is not a reliable indicator of the biological effects of exogenous GH treatment in genetically and pharmacologically unmodified individuals (Bielohuby et al., 2011). IGF1 plays an essential role in the body growth, especially the postnatal life, and is involved in many cell processes, including cell proliferation, cell differentiation, and cell apoptosis through the IGF1 receptor (IGF1R) and the subsequent activation of MAPK/ERK1/2 and PI3K/Akt signaling pathways (Yamauchi and Pessin, 1994; Grey et al., 2003). IGF1 treatment may up-regulate the IGF1R expression and the activation of PI3K/Akt and MAPK/ERK1/2 pathways mediated by IGF1R (Matà et al., 2016). In addition, the role of IGF1 depends on at least six binding proteins (IGFBP1-5, 7). In both circulation and local tissues, 90% of IGF1 binds to IGFBP3 to inhibit its binding to IGF1R, thereby prolonging the half-life of the IGF1 (Brahmkhatri et al., 2015). On the other side, serum IGF1 exerts a negative feedback on GH production, by directly inhibiting GH secretion from pituitary gland and indirectly promoting SS or inhibiting GHRH secretion (Tannenbaum et al., 1983).

## Role of GH-IGF1 Signaling in Variant Cancers

GH-IGF1 axis has many regulatory functions in various tissues and cells, and thus the effects of GH-IGF1 axis on cancer progression has attracted considerable interest recently. GH-IGF1 axis dysregulation enhances the synergistic effect of the promotion of uncontrolled cell proliferation, cell migration and invasion, thus promoting cancer initiation and metastasis (Dehkhoda et al., 2018). In the past, body height is considered as a biomarker action of GH and IGF1. Interestingly, it is reported that body height associated with GH and IGF1 levels also related to cancer risk, individuals taller than 175 cm have a 20% higher risk of developing prostate cancer than those shorter than 160 cm, a 20–60% higher risk of developing colorectal cancer, and have 22% higher risk of developing breast cancer (Gunnell et al., 2001; Lango Allen et al., 2010). In a word, GH and IGF1 levels are associated with cancer risk.

Growth hormone excess leads to several variants of pituitary tumor (Akirov and Asa, 2019). After a cranial radiation therapy induced GH deficiency (GHD), 4.7% GH-treated survivors developed subsequent neoplasms of the central nervous system, while only 1.7% developed without GH treatment (Patterson et al., 2014). In addition to the context of hypopituitarism, GHD also occurred in the genetic models. Genetic isolated GHD (IGHD) can be an alternative way to assess the biological impacts of GH (Aguiar-Oliveira and Salvatori, 2021). Individuals with GHR deficiency (GHRD) caused by genetic mutations in the displayed *GHR* gene and congenital IGF1 appear to be protected against the development of neoplasms. This protection, although also well documented, is not absolute in IGHD, GHD associated with multiple pituitary defects and GHRH receptor deficiency (Shevah and Laron, 2007; Guevara-Aguirre et al., 2011; Steuerman et al., 2011; Marinho et al., 2018).

### Role of GH Signaling in Cancers

**FIGURE 2:**
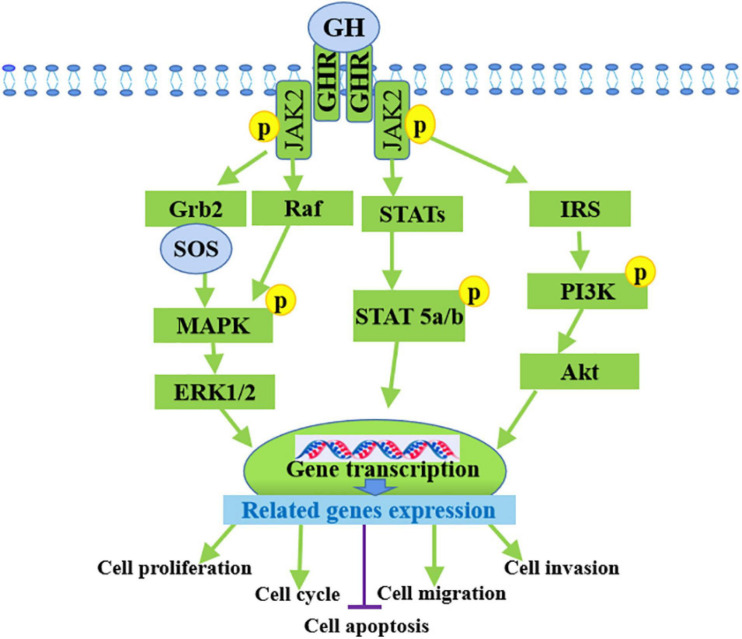
Mechanism of GH in promoting cancer progression by binding to GHR. GH activates multiple signaling pathways by binding to its receptor GHR to promote the development of various cancers through the inducing of cell proliferation, invasion, migration and inhibiting cell apoptosis.

Growth hormone and its mediated signaling pathways are involved in the development of multiple cancer types, particularly colon, breast, and endometrial cancers according to *in vitro*, *in vivo*, and epidemiological analyses. GH accelerates oncogenic signaling for common oncogenic processes and tumor development, including proliferation, migration, invasion (Brunet-Dunand et al., 2009), metastasis (Brittain et al., 2017), and epithelial-to-mesenchymal transition (EMT) (Mukhina et al., 2004), and prevents apoptosis by exerting autocrine or paracrine effects on cancer cell behavior and neighboring cells within the tumor microenvironments (Perry et al., 2017). In endometrial cancer, autocrine hGH enhances the oncogenic characteristics *in vitro* and promotes the growth of RL95-2 tumors when GHR is overexpressed *in vivo* (Pandey et al., 2008). In addition, hGH expression level and is closely correlated with histopathological features, such as the higher tumor grade, myometrial invasion, and ovarian metastases, as well as poor prognosis (Slater et al., 2006; Wu et al., 2011). Research indicated that GH promoted JAK2 and GHR tyrosine phosphorylation and STAT5 activation in prostate cancer cells (Gan et al., 2014). Silencing GHR sensitized hepatocarcinoma cells to sorafenib, and may inactivated the PI3K/Akt and ERK1/2 signaling pathways (Figure 2). Hence, the efficacy of sorafenib in liver cancer can be enhanced by using GHR as a target (Gao et al., 2020). *In vitro* and *in vivo* studies have evaluated the biological functions of GHR in breast cancer by silencing GHR and showed that GHR reduction leads to the inhibition of cell proliferation, the cell-cycle arrest in G1-S phase transition, tumor growth, and cell apoptosis induction of breast cancer cell lines, speculating these effects might be due to the inhibition of the Raf/MEK/ERK signaling pathway (Zhu et al., 2020). A recent study on humans and mice demonstrated that disrupted GH signaling was associated with elevated p53 levels in colon tissue, suggesting GH may act as a tumor promoter by suppressing gene transcription of p53, PTEN, and APC levels (Chesnokova et al., 2016). A small number of GHR antagonists have been used in an oncology settings in preclinical studies and the growth-inhibiting effects have been reported in colon, breast, and meningioma tumor xenografts, which suggested that GHR antagonism as a monotherapy is efficient in some tumor types (Friend, 2001; McCutcheon et al., 2001; Dagnaes-Hansen et al., 2004; Divisova et al., 2006). The activation of GH and its receptor GHR can promote the development of various cancers by enhancing cell proliferation, invasion, and migration and inhibiting cell apoptosis. These processes occur through the regulated expression of related genes.

### Role of IGF1 Signaling in Cancers

Numerous studies have demonstrated that IGF1 signaling is involved in tumor growth and is a prognostic factor for different cancer types (Papa et al., 1993; Ouban et al., 2003; Yanochko and Eckhart, 2006; Kim et al., 2007).

**FIGURE 3:**
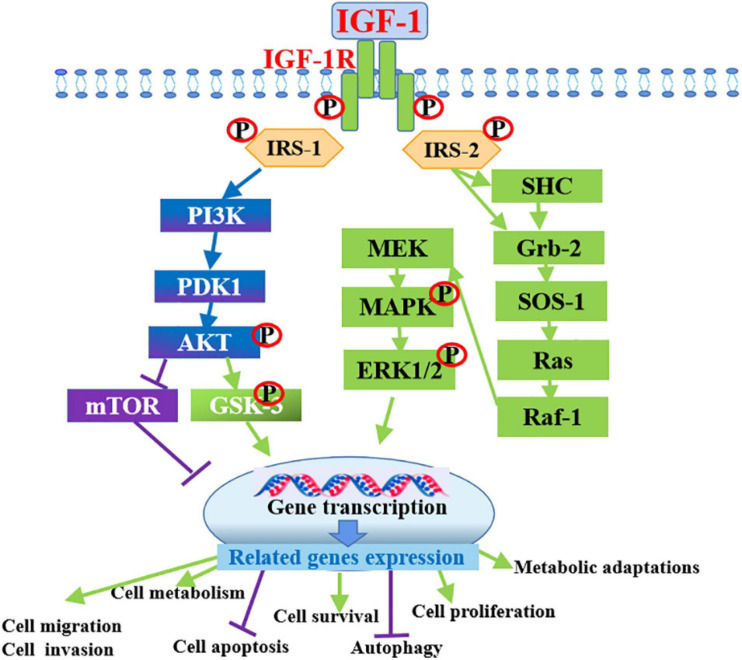
Intracellular mechanism of IGF-1 in promoting cancer progression by binding to IGF-1R. IGF-1 activates PI3K/Akt and Ras/MAPK/ERK1/2 signaling pathways by binding to its receptor IGF-1R to promote the development of various cancers through the inducing of cell survival, cell metabolism, invasion, migration and inhibiting cell apoptosis and autophagy.

Given that IGFs are involved in the regulation of cell metastasis, their roles in the metastasis and proliferation of cancer cells have been a focus of many studies in recent decades. Researchers have attempted to establish the associations between serum IGFs levels and cancer risk. The circulating level of IGF1 coupled with IGFBPs has been correlated with the risk of developing various cancers, including breast, lung, colon, and prostate cancers through human epidemiological studies (Chan et al., 1998; Hankinson et al., 1998; Ma et al., 1999; Yu et al., 1999; Shi et al., 2001). A nested case-control study in the prospective prostate cancer screening trial was conducted to examine the associations between IGF1/IGFBP3 levels and the risk of prostate cancer, in this trial, a total of 727 incident prostate cancer cases and 887 matched controls were selected for analyses, the result indicated that the bioavailability of IGF1 was associated with risk for aggressive prostate cancer in obese men (Weiss et al., 2007). Circulating IGF1 level plays a significant role as a risk factor for the onset and development of various tumors by the increase of neoplastic cell proliferation, such as lung, breast, ovarian and colorectal cancers (Myal et al., 1984; Wu et al., 2003). The PI3K pathway activated by IGF1R usually alters in cancer cells (Cairns et al., 2011), which not only provides anti-apoptotic and mitogenic signals but also affects cancer cell metabolism, in this process, Akt, the downstream target of PI3K, has been shown to stimulate the glycolytic pathway, favoring energy production in the tumors (Plas and Thompson, 2005). mTOR signaling factors downstream of IGF1R play key roles in the regulation of cancer cell metabolism, lipid and protein synthesis and they are responsible for several metabolic adaptations (Guertin and Sabatini, 2007). *In vitro* studies, over-expression of the IGF1R has been identified as a typical hallmark of many types of tumors (Werner et al., 2012). Cells derived from *IGF1R^–/–^* (complete deficiency of IGF1R) animals do not undergo malignant transformation when exposed to oncogenes (Sell et al., 1993; Sell et al., 1994; Figure 3).

In addition, many studies have confirmed that genetic mutations in the *IGF1* gene are associated with the occurrence and development of cancer. The polymorphisms of the *IGF1* gene, namely, rs12423791, rs1019731, rs5742632, rs2033178, and rs2373722, are associated with the risk of colon cancer in Tunisian population (Dhifallah et al., 2019). Statistically significant association was detected between short *IGF1* CA repeats and increased risk for colorectal cancer in hereditary non-polyposis colorectal cancer (Zecevic et al., 2006). The reasons for these correlations may be the changes in gene expression levels caused by the polymorphisms.

In terms of mechanism, the cross-talk between IGF1R and carcinogenic factors, including integrins, focal adhesion kinase, and the RACK1 scaffolding protein enhanced the motility and migration of cells (Chitnis et al., 2008). IGF1R stimulates tumor metastasis and the secretion of invasion factors to the extracellular matrix (Tressler et al., 1993; Hajjar and Krishnan, 1999), which is independent of the main signaling pathways related to IGF1 (Zhao et al., 2003).

## Role of GH-IGF1 Signaling Pathways on Therapy Resistance in Cancer Radiotherapy

Radiotherapy through ionizing radiations, such as X-rays and γ-rays, remains one of the most important cancer treatment modalities and is used for approximately 50% of all cancer patients with varying success. Radiotherapy inhibits tumor growth by generally inducing tumor cell apoptosis, necrosis, mitotic catastrophe, autophagy, or senescence through inflicting extensive DNA damage, including DNA-protein crosslinking, or transiently increasing the levels of cytotoxic reactive oxygen species (ROS) (Baskar et al., 2012; Chen and Kuo, 2017). Radiotherapy has been widely used in tumors such as skin, cervical, head-and-neck, laryngeal, nasopharyngeal, prostate, and breast cancer, however, many types of tumors, including glioblastoma, soft-tissue cancers as well as non-small cell lung cancer are less sensitive to radiotherapy due to their intrinsic resistance. Moreover, the acquired resistance of sensitive tissues to radiotherapy is also common (Barker et al., 2015; Kim et al., 2015). Therefore, the development of resistance to radiotherapy is one of the toughest challenges in disease management. Based on the roles of GH and IGF1 signaling pathways on anti-apoptotic and mitogenic, the abnormally high expression of GH and IGF1 in cancer cells may lead to their resistance to radiotherapy.

### Irradiation-Induced GH Deficiency

Subtle and frank neuroendocrine abnormalities occur following radiation damage to the hypothalamic-pituitary axis. Anterior-pituitary hormone deficiencies represent the most common complications of successful cancers therapy in both children and adults by radiation, such as brain tumors, face and neck tumors, skull base tumors (Darzy, 2009). GHD is the most frequent complication and usually the only overt manifestation of neuroendocrine injury in the vast majority of irradiated patients (Darzy and Shalet, 2006).

GHD is more common after radiotherapy for treatment of childhood cancer (Sfeir et al., 2018). Radiotherapy for childhood cancer usually includes two irradiation methods. One is cranial or craniospinal irradiation for brain, nasopharyngeal, and orbital tumors and central nervous system leukemia, and the other is total body irradiation as a preparation for bone marrow transplantation. Nearly 100% of children treated with radiation doses in excess of 30 Gy have blunted GH responses to insulin tolerance tests (Richards et al., 1991). In the low-dose cranial irradiation (18–24 Gy), GHD is often the only deficiency hormone in pituitary gland (Chemaitilly and Sklar, 2010; Torino et al., 2013), low radiation doses lead to variable GHD levels in patients with leukemia or brain tumors (Littley et al., 1989; Lam et al., 1991; Constine et al., 1993). The severity and speed of onset of radiation-induced GHD are dose-dependent, and the incidence also increases over time elapsed after irradiation. Isolated GHD (peak GH response < 9 mU/l) can be observed after 18-24 Gy cranial irradiation (Shalet et al., 1979; Kirk et al., 1987; Costin, 1988; Brennan et al., 1998) more frequently in children than in adults (Brauner et al., 1986). In children who received a radiation dose of at least 2700 cGy (Brauner et al., 1985) for brain tumors over 3 weeks (Shalet et al., 1978), the majority showed impaired GH secretion within 1 or 2 years. In addition, a subtle defect in GH secretion occurs in head-irradiated rats (Brauner et al., 1985). Chrousos et al. (1982) assessed decreased GH secretion (0.1 U/kg) 1 year after over 2 weeks of cranial irradiation in the monkeys (Chrousos et al., 1982).

### Irradiation-Induced Growth Failure

Growth hormone is the most important factor in determining the height of a child during the growth and development stage. Frequently, brain tumor and its therapy are endocrinologically devastating for children, and the most common residua are growth failure or short stature, which may be caused by the high rate of GHD in children after cranial radiation and radiation effects on skeletal growth after spinal or whole-body radiation treatment (Brauner et al., 1989). Children who are treated with cranial radiation generally undergo a decrement in body height velocity during and after the first year of remission induction (Schriock et al., 1991). Significant limitation in the growth of the upper segment was observed in patients who underwent spinal radiation in addition to cranial radiation for their central nervous system tumors (Clayton and Shalet, 1991). Two-days-male rats received 600 cGy X-irradiation to the head showed significantly stunted body weight and tail length beginning prior to weaning and lasting throughout the period (64 days) of observation (Mosier et al., 1985).

### Response of IGF1 Signaling Pathway to Radiation

Heavy-ion ^56^Fe radiation led to increased serum IGF1 levels 2 months after exposure and decreased IGFBP-3 levels in serum along with increased IGF1R in tissues (Suman et al., 2016). Fractionated radiation induces an increase in IGF1 secretion level and gradually up-regulates IGF1R expression in cancer stem cells (GSCs) (Osuka et al., 2013; Osuka and Van Meir, 2017). One of the mechanisms maybe the radiation-induced exosome export of miR-603 and the simultaneously up-regulation of IGF1 and IGF1R expression (Ramakrishnan et al., 2020). IGF1R up-regulation exerts a dual radio-protective effect. In the resting state, continuous IGF1 stimulation ultimately induced down-regulation of Akt or ERK and Foxo3a activation, thereby inhibiting proliferation and enhancing self-renewal. By contrast, after acute radiation, the abundance of IGF1R and increased secretion of IGF1 promoted a rapid shift from a latent state toward the activation of the Akt survival signaling, protecting GSCs from radiation toxicity (Osuka et al., 2013). Ionizing radiation may lead to changes in the serum levels of cancer-related hormones and proteins in cancer-free women, including IGF1 (Grant et al., 2011). Paracrine IGF1/IGF1R signaling initiated by radiotherapy-activated cancer-associated fibroblasts promotes colorectal cancer progression (Tommelein et al., 2018), and the radiation-induced secretion of IGF1 in human fibroblasts up-regulated IGF1R/Akt signaling in bystander cells (Ivanov et al., 2010). Results demonstrated for the first time in the gastrointestinal that ionizing radiation persistently increases IGF1 and activates downstream PI3K/Akt and JAK2 signaling pathways, which may contribute to gastrointestinal functional alterations and carcinogenesis (Suman et al., 2015).

### Effects of GH Signaling on Therapy Resistance in Cancer Radiotherapy

*In vitro* studies, researchers have clearly shown that GH treatment conferred radio-resistance in tumor cells. In addition, GH may be a radio-protective agent (Tekin et al., 2006). Autocrine GH enhances cell viability, clonogenic survival, and DNA repair in breast and endometrial cancers after ionizing radiation (Bougen et al., 2012). Conversely, the functional inhibition of GH signaling using a specific GH receptor antagonist in endometrial cancer cells sensitizes cells to ionizing radiation-induced cell death and enhances the induction of DNA damage (Bougen et al., 2012). Combining recombinant GH with radiation increases clonogenic survival and reduces DNA damage in a colorectal cancer cell line (Wu et al., 2014), whereas the over-expression of GHR in rectal cancer is predictive factor for tumor response to preoperative radiotherapy (Wu et al., 2006). GH treated-breast cancer cells MDA-MB-435S, T47D, and endometrial cancer cell RL95-2 show significantly reduced DNA damage and heightened clonogenic survival post-irradiation (Bougen et al., 2012). The protective effect of GH on radiation in radiated GHR-expressing human colorectal cancer cell HCT-8 treated with hGH or GHR antibody has been demonstrated on the basis of cell survival rate and DNA damage detection (Wu et al., 2014).

A *in vivo* study on pre-operative biopsy and post-irradiation specimens in 98 patients of rectal cancer involving human cancer patients found that increased GHR expression level was associated with poor response to radiation treatment, suggesting that GHR-antagonism can actually improve rectal cancer sensitivity to radiotherapy (Wu et al., 2006). Another study on nude mice with RL95-2 cell xenografts showed reduced growth and anti-vascular effects in the GHR antagonism group when γ-irradiated with or without GHR antagonism injections (Evans et al., 2016). Collectively, the results *in vitro* and vivo confirm that GH and the signaling pathways provide protection against damage due to radiotherapy in human cancers and GH antagonism sensitizes cancer to radiotherapy.

### Protective Effect of IGF1 on Radiation Damage in Cancer Radiotherapy

Signaling pathways activated by IGF1/IGF1R respond to radiotherapy in several types of cancer (Buck et al., 2008; Dallas et al., 2009; Eckstein et al., 2009; Sharma et al., 2010). Increased expression of IGF1R confers radio-resistance to cells (Trojanek et al., 2003; Turney et al., 2012). The up-regulation of IGF1R signaling promotes radio-resistance in several types of solid tumors (Turner et al., 1997; Bartucci et al., 2001; Macaulay et al., 2001). In bulk tumor cells, the IGF1R-induced activation of PI3K-Akt signaling results in radio-resistance by inhibiting cell apoptosis and promoting cell survival (Robbins et al., 1992; Valenciano et al., 2012). On the contrary, depletion of IGF1R delayed the repair of radiation-induced DNA double strand breaks (Trojanek et al., 2003). A recent study also shows that blocking IGF1R-mediated signaling reduces cell proliferation and increases cells sensitivity to the destructive effects of ionizing radiation mediated by down-regulation of BRCA2, a mediator of HR, suggesting that IGF1R is a target for enhancing tumor sensitivity to radiotherapy (Venkatachalam et al., 2017). The treatment of tumors formed by the radio-resistant GSCs with an IGF1R inhibitor results in a marked increase in radio sensitivity, suggesting that the blockade of IGF1R signaling is an effective strategy to reverse radio-resistance (Osuka et al., 2013).

In addition, *in vitro* study has indicated local IGF1 can enhance the radiation resistance to prevent cell death (Pal et al., 2018). Abdominal irradiation induces intestinal epithelial cell apoptosis and that treatment with GH or IGF1 treatment can significantly inhibit this effect (Mylonas et al., 2000), while there is study also suggests that GH shows radioprotective effect through increasing local IGF1 level and counteracting cell apoptosis (Mahran et al., 2015).

### Multiple Mechanisms of Resistance to Radiotherapy by GH and IGF1

Radiation resistance leads to reduced effectiveness of radiotherapy in many patients and may cause metastasis and cancer recurrence frequently. Thus, understanding the molecular mechanisms that cause radio-resistance in cancers may be useful in improving adjuvant treatments for enhancing the efficacy of radiotherapy. GH and IGF1 both have significant effects on radiotherapy resistance and post-radiotherapy recovery (Redelman et al., 2008; Bougen et al., 2012). Therefore, we summarized the multiple mechanisms of developing resistance to radiotherapy.

#### Adaptation to Radiation Through Oxidative Stress Overloading

Reactive oxygen species level and the balance of ROS level in cancer cells are important in radio-resistance (Reuter et al., 2010). Therefore, radiation tends to induce ROS levels and adaptive antioxidant defense systems, which may lead to radio-resistance (Riley, 1994; Guo et al., 2003; Qu et al., 2010). Persistent oxidative stress regulates many transcription factors/activators, such as NF-κB and p53, thereby influencing the cell cycle and DNA repair signaling pathways (Reuter et al., 2010).

#### DNA Damage Repair

Ionizing radiation damages biological macromolecules directly or indirectly. The most important one is DNA damage. DSB is the deadliest of DNA damage. If not repaired, it would lead to chromosome aberration, cell transformation, and apoptosis. DSBs can be repaired by NHEJ or HR, respectively. Among them, NHEJ reassembles a broken DNA end under DNA-PKcs, while HR is a high fidelity repair mechanism activated by ATM activation (Williams et al., 2007; Ciccia and Elledge, 2010), and mainly acts on the S and G2 stages of the cell cycle. The repair pattern needs to be mediated by BRCA1, BRCA2, RAD51, and 53BP1. IGF1 signaling promotes DNA repair by activating ATM and DNA-PKcs. Due to radiotherapy triggers cell cycle checkpoints to block cells from entering mitosis, the apparent over-expression of ATM/ATR could simply reflect changes in the radio-resistance activity of cancer cells. Besides, PARP-1, which is involved in DNA damage repair and the regulation of related transcription factors.

#### Increased Cell Adhesion to Extracellular Matrix (ECM)

Extracellular matrix is a non-cellular highly dynamic essential support structure within tissues (Ramteke et al., 2015). Up-regulation of a number of collagen genes is associated with radio-resistance (Seregard et al., 2013). Increasing type-IV collagen-mediated signaling drastically increases metastases in multiple tumor types, especially those with markedly higher IGF1R expression levels (Lalonde et al., 2014). While GH is known to increase both collagen synthesis in human subjects (Chughtai et al., 2011) and collagen degrading related genes and TGF-activating matrix metalloproteinases (Deng et al., 2011) in tumors.

In nature, unlimited proliferation and shorter cell cycle are essential differences between cancer cells and normal cells, and one of the intrinsic mechanisms of radiotherapy resistance is cell proliferation inhibiting and apoptosis promoting. While the primary function of GH and IGF1 and their mediated signaling pathways is promoting cell proliferation. Therefore, both the cancer protection mechanism and the radio-resistance mechanism of GH and IGF1 are via their role in regulating cell proliferation and apoptosis.

**FIGURE 4:**
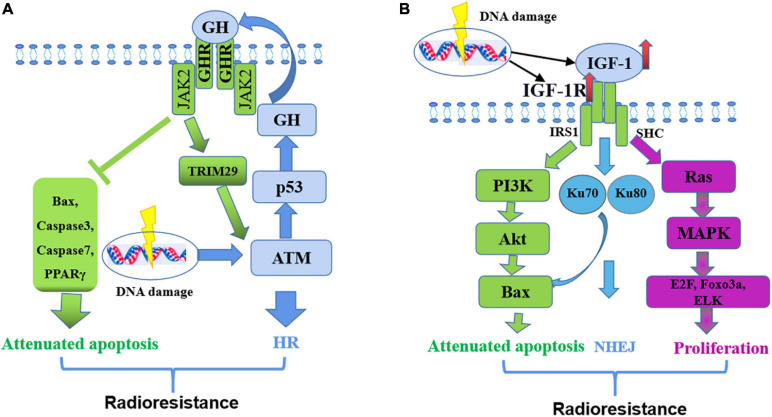
Mechanism of GH-IGF-1 signaling in resistance to radiotherapy. **(A)** GH regulates radio-resistance by inducing HR pathway to repair DNA damage and decreasing pro-apoptotic molecules. **(B)** IGF-1 regulates radio-resistance by promoting cell proliferation, inducing NHEJ pathway to repair DNA damage and attenuating cell apoptosis.

Growth hormone mediated therapy resistance in human cancers undergoes the following steps (Basu and Kopchick, 2019): radiotherapeutic interventions cause DNA damage in tumor cells and induce ATM activation (Ciccia and Elledge, 2010); ATM mediates p53 production; P53 directly increases GH production, endocrine GH promotes the expression of ATM through JAK2-activated TRIM29, and next regulates HR pathway to repair DNA damage (Sho et al., 2011); GH exerts autocrine/paracrine effects by binding to GHR in the same or a neighboring cell surface and initiating JAK2- and SRC-mediated signaling cascades, which decrease pro-apoptotic molecules (Bax and PPARγ) and suppress caspase protein activation (Basu and Kopchick, 2019), thereby preventing cell death and providing resistance to therapy (Figure 4A).

IGF1 mediates therapy resistance in human cancers simply through the following steps: Firstly, radiotherapy increases the expression of IGF1 and IGF1R (Osuka and Van Meir, 2017); IGF1 combines with IGF1R, activates the Ras/MEK signaling pathway, and promotes the expression of transcription factors, such as E2F, Foxo3a, and ELK (Reuter et al., 2010), thereby promoting cell proliferation; At the same time, IGF1R activates the PI3K/Akt signaling pathway, promotes Bcl-2/Bax expression and inhibits cell apoptosis (Basu and Kopchick, 2019); Activated IGF1R also increases Ku70 and Ku80 expression levels and thereby promotes DNA repair by NHEJ (Katayama et al., 2007) (Figure 4B).

## The Favorable Impact of Gh-Igf1 on Radiation Damage Repair in Adjacent Tissues

Radiotherapy could kill tumor cells and improve the survival rate of patients, but meanwhile cause inevitable damage to adjacent tissues in the radiotherapy area unavoidable. We have summarized the resistance of GH and IGF1 signaling pathways to radiotherapy, which is not conducive to treatment, but a large number of studies have shown that they have recovery effect on the damage of adjacent tissues post-radiotherapy.

### Effects of GH on the Damage Repair of Adjacent Tissues Induced by Radiotherapy

Reduced cell apoptosis, related gene expression levels and caspase proteins are presented in GH over-expressed EL4 T-cell lymphomas when treated with methyl methanesulfonate (Arnold and Weigent, 2004). GH induces DNA-damage in by the feedback inhibition of p53 (Chesnokova et al., 2013, 2016, 2019). Cell death of irradiated peripheral blood lymphocytes is rescued after hGH treatment by activating Bcl2 and restoring the cytokine secretory profile (Lempereur et al., 2003). Non-human primates exhibit similar protective effects by GH (Prieto et al., 1998). Adult male Wistar rats show improved rescue from an abdominal mucosal lesion caused by a lethal radiation dose after GH treated for 7 days (Lempereur et al., 2003). BDIX rats with colon tumor xenografts treated with radiation exhibit a GH-induced apoptosis decrease and preferential protection in non-tumor intestinal cells (Morante et al., 2003). The anti-apoptotic effects of rhGH are validated in irradiated BALB/c mice treated for 35 days irradiation, and rhGH treatment significantly restores hematologic and immune (Chen et al., 2010). Interestingly, IGF1 exhibits an identical anti-apoptotic effect on irradiated BALB/c mice, indicating that part of the radioprotection of GH might be mediated via IGF1 (Zhou et al., 2013).

### Effects of IGF1 on the Damage Repair of Adjacent Tissues Induced by Radiotherapy

Report has indicated that *in vivo* IGF1 increased epithelial growth and enhanced crypt regeneration in mice intestine after radiation via distinct regulatory pathways. And in the intestinal stem cells isolated from the uninjured and regenerating intestines, IGF1 increases the quantities of Sox9-Low and Sox9-High cells and the percentage of these cells in M-phase (Van Landeghem et al., 2015). When IGF1 is absent, the activation of IGF1R and the downstream phosphorylate Akt is lower after irradiation, resulting in low proteoglycan synthesis. In addition, radiation induces active degradation of matrix by impairing IGF1 signaling in human or pig cartilage and chondrocytes. Furthermore, an imbalance may exist in the PI3K/Akt pathway and MAPK pathway activated by IGF1R following irradiation in chondrocytes (Willey et al., 2013). The activated IGF1R influences DNA damage repair in irradiated skin keratinocytes. Specifically, the rate of DNA damage repair following irradiation is significantly reduced when the activation of IGF1R was absent, both in immortalized human keratinocytes and in primary human keratinocytes. Furthermore, DNA damage repair is significantly suppressed using either vitro explant cultures or *in vivo* xenograft models to inhibit IGF1R activity in human skin. Similarly, the primary keratinocytes with an inactivated IGF1R exhibited lower stable levels of nucleotide excision repair mRNAs (Loesch et al., 2016).

## Role of GH-IGF1 Signaling in DNA Damage Repair

Cells are continuously faced with endogenous stress or exogenous stress, for example, radiation can ultimately lead to DNA damage, even genomic integrity. To limit genomic instability, the cells have a series of repair proteins that engage the appropriate DNA repair pathways, and then produce some damage repair effects. DDR pathways are activated to repair DNA. Hormones and their cognate receptors play an important role in DNA damage regulation.

### The Role of GH in Promoting Growth Rate and DNA Damage Repair

Previously we have introduced that the radiation may lead to short stature by GHD. Six children suffering from radiation-induced GHD treated with GH between 3 and 10 years after cranial irradiation exhibit a mean growth during the pretreatment year of 3.7 cm and during the first year of GH therapy is 7.9 cm, indicating the recovery effect of GH on radiation damage (Shalet et al., 1981). The same recovery effect is also exist in other children with radiation-induced GHD (Perry-Keene et al., 1976; Richards et al., 1976; Romshe et al., 1984). However, children do not receive GH therapy have dysplastic in height and have trend to be extremely short stature. GHD is much worse in children who received radiation for brain tumors than for the other tumors (Brauner et al., 1989; Clarson and Del Maestro, 1999). During the children’s transition to adulthood after suffering from radiotherapy, they also need GH treatment to maximize their bone mineral density and prevent osteoporosis (Murray et al., 1999; Brennan et al., 2005). Of course, radiation-induced GHD in adults might be associated with signs and symptoms of adult GH deficiency syndrome, including reduced basal metabolism, blood volume, cardiac output, and glomerular filtration rate, thus impairing life quality (Bengtsson and Johannsson, 2000). After GH therapy, the life quality of these patients with pituitary tumors is improved to an extent (Murray et al., 2002). As a consequence, it is important for the robust diagnosis of radiation-induced GHD, ensuring the GH replacement therapy can be implemented at the right time.

Studies addressed that hGH had a radio-protective effect both *in vivo* and *in vitro* possibly due to DNA repairing mechanisms. *In vivo*, GH reduces mortality and bacterial translocation in irradiation rats (Gómez-de-Segura et al., 1998), and *in vitro*, GH protects against radiotherapy-induced cell death by DNA repairing (Madrid et al., 2002).

The repair effects of GH on DNA damage are cell type-dependent. GH synchronously increases cell proliferation and increase DNA damage repair in CHO cells (Madrid et al., 2002), and also protects human breast cancer cells from the DNA-damaging effects by cytotoxic drugs (Zatelli et al., 2009). GH promotes DNA damage repair by reducing apoptosis and increasing drug resistance in human endometrial cancer cell AN3CA and breast cancer cell MCF-7 (Zatelli et al., 2009; Gentilin et al., 2017). CHO cells with over-expressed GHR increased DNA repair and protected cells from radiation-induced death after treating by GH (Madrid et al., 2002). Colon adenocarcinoma HCT-8 cells treated by hGH exhibits reduced radiation-induced DNA damage by up-regulating GADD45 and APEN genes (Wu et al., 2014). Autocrine hGH is shown to increase clonogenic survival and attenuate radiation-induced or mitomycin-induced DNA damage in human mammary cells and endometrial carcinoma cells. The protective and DNA damage repair effects of GH are generally mediated by the JAK2 and c-Src family activated by GHR and the GH induced DNA damage repair genes, including BRCA1, BRCA2.

However, in some special circumstances or at special time points, treatment with GH may also increase DNA damage or decrease DNA damage repair, such as exposing GH to the GHD Lewis dwarf rats and Snell dwarf mice in prepubertal results in decreased DNA repair by leading to dysregulation of DNA damage proteins (Podlutsky et al., 2017). Besides, in non-transformed cells and tissues, GH appears to induce DNA damage. Recently report address that GH inhibits DNA damage repair by suppressing ATM and DNA-PKcs activities in colon cells, leading to decreased DNA repair by both HR and NHEJ (Chesnokova et al., 2019). *In vivo*, injecting hGH to human subjects for 6 weeks results in increased lymphocyte DNA damage, and the damage can last for a long time after hGH withdrawal (Fantini et al., 2017). Patients with pituitary adenomas also have increased DNA damage in peripheral blood lymphocytes due to the GH excess secretion (Bayram et al., 2014). In general, GH induces DNA damage by suppressing ATM kinase activity and decreasing phosphorylation of key DDR proteins, such as p53, Chk2, and γH2AX.

### IGF1 Promotes DNA Damage Repair of DNA DBSs

IGF1 and IGF1R are necessary for nucleotide excision repair (Auclair et al., 2008; Loesch et al., 2016). The role of IGF1R in DDR includes promotion of DNA repair. In the skin, IGF1R signaling regulates DNA repair through downstream targets PI3K/Akt (Kemp et al., 2017a, b) or through the ATR-Chk1 kinase pathway (Strozyk and Kulms, 2013). Over-expressed IGF1R promotes DNA repair in primary human lung fibroblasts, several human cancer cells and irradiated salivary glands (Meyer et al., 2017). Suppressing IGF1R reduces DNA damage repair by decreasing ATM phosphorylation, which played a critical role in HR and NHEJ (Turney et al., 2012; Chitnis et al., 2014).

### Mechanisms of GH and IGF1 Influences DNA Repair

Ataxia telangiectasia mutated activation is critical in the response of GH to DNA damage repair, which can be regulated by different pathways. Importantly, DNA damage induces DNA-binding complex MRN (Mre11/Rad50/Nbs1), which is required for ATM activation (Lee and Paull, 2007), and GH rapidly dephosphorylates Mre11 and Rad50 in 3T3-F442A preadipocytes according to the phosphoproteomics results. In this process, DNA damage promotes GH induced TRIM29 binds (Sho et al., 2011) and ATM activation. by activating molecules related to cell cycle checkpoint, apoptosis, senescence, chromatin structure alterations, and DNA repair. (Jette and Lees-Miller, 2015; Blackford and Jackson, 2017). MRN-dependent ATM stimulation triggered phosphorylation of DNA-PKcs to induce NHEJ and HR (Jazayeri et al., 2006).

**FIGURE 5:**
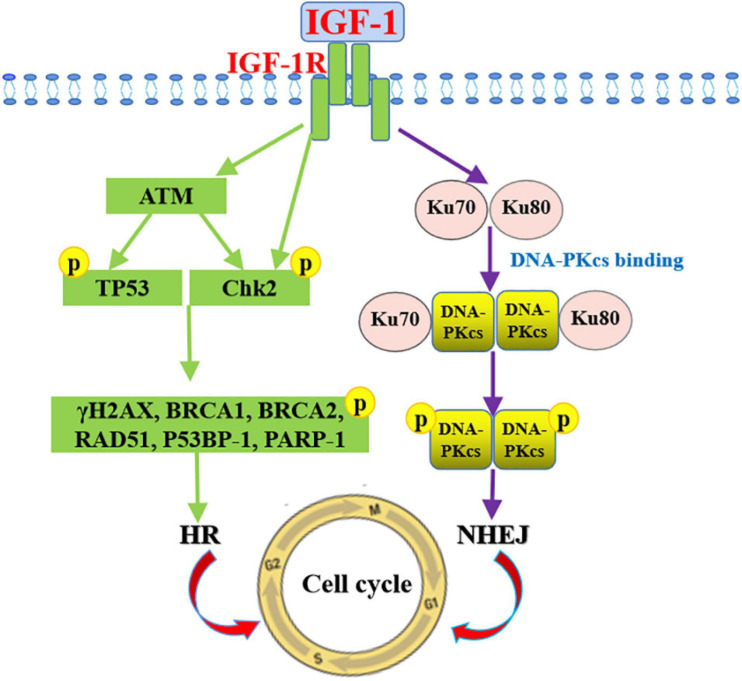
Mechanism of IGF-1 signaling in promoting DNA damage repair. Signaling pathways mediated by IGF-1/IGF-1R promotes DNA repair by NHEJ through increasing phosphorylated DNA-PKcs and Ku70/Ku80 expressions and by HR through inducing phosphorylated ATM, γH2AX, p53BP-1, and PARP-1 expression levels.

The mechanisms by which IGF1/IGF1R influences DNA repair have been reported. IGF1 stimulation could induce DNA repair by HR in MCF7 cells (Trojanek et al., 2003; Yang et al., 2005). IGF1 indirectly increases DNA repair by 50% through increasing the p53 dependence in the p21, while inhibition IGF1R by siRNA in human prostate cancer cells enhances the sensitivity of ionizing radiation to SSBs and DSBs (Clark et al., 2005). DNA repair in cells lacking IGF1R decreases by 52%, confirming the role of IGF1 signaling in DNA repair (Turney et al., 2012). On the other hand, DNA damage-induced ATM activates IGF1/IGF1R in mouse embryonic fibroblasts (Goetz et al., 2011). When IGF1R is suppressed by siRNA, mouse melanoma cells fail to induce ATM kinase activity after irradiation (Macaulay et al., 2001), suggesting that IGF1R plays a critical role in mediating ATM. In addition, suppressing IGF1R blocks the repair of DSBs in human prostate cancer DU145 and PC3 cells and makes them sensitive to ionizing radiation (Clark et al., 2005). Besides, suppressing of IGF1R both leads to ATM kinase inactivation and inhibited DNA-PKcs phosphorylation, which are involved in the DNA repair of NHEJ (Chitnis et al., 2014). IGF1R inhibitors inhibits radiation-induced DNA damage repair by prolonging the expression of phosphorylated histone γH2AX, and interfering with the Ku-DNA binding and Ku70/Ku80 expressions (Katayama et al., 2007). To sum up, there is a direct relationship between radiation-induced DNA damage repair and IGF1/IGF1R pathways. Inhibiting the pathways improves the sensitivity to irradiation and reduces the expression of phosphorylated ATM, γH2AX, p53BP-1, DNA-PKcs, and PARP-1 related to the DNA repair pathways (Figure 5).

## Concluding Remarks

Radiation exposure to key endocrine organs (e.g., hypothalamus, pituitary, thyroid, and gonads) places cancer survivors at the highest risk of developing an endocrine abnormality over time, these endocrinopathies can develop decades following cancer treatment (Sklar et al., 2018), and are also observed in survivors treated with radiation to the head, neck, or pelvis (Chemaitilly et al., 2018). GH and IGF1, as critical endocrine factors are involved in promoting cell proliferation and inhibiting cell apoptosis in cancer cells and normal cells. Based on this, they play key roles both in the occurrence and development of cancers and in the repair of normal tissue damage. In radiotherapy for cancer, radiation resistance induced by GH and IGF1 signaling reduces the effectiveness of radiotherapy in many patients and may cause metastasis and cancer recurrence frequently. Therefore, in theory, giving GH or IGF1 antagonist treatment in cancer radiotherapy may increase the radiation sensitivity of cancer cells, and promote the effectiveness of radiotherapy. However, considering that the dose and time of administration may affect the killing efficiency and the recurrence of different cancer cells by radiotherapy, further research through *in vivo* and *in vitro* experiments is necessary. Similarly, dosage and time of administration of GH and IGF1 or their analogs for the repair of adjacent tissue damage after radiotherapy need to be determined according to the type of tumor cells for the most ideal effect. For instance, for children with tumors, GH treatment usually is given more than 1 year after radiotherapy to improve GHD and short stature caused by radiotherapy and prevent cancer recurrence. In terms of mechanism, DNA damage repair is the most critical reason for the radiotherapy resistance of GH and IGF1 in cancer cells and damage repair effect on adjacent tissues. However, *in vivo* studies on the effects of GH and IGF1 on DNA damage repair after radiotherapy are few, and a large number of animal experiments are still needed.

## Author Contributions

YC and LH designed the ideas. WL, RG, and XW helped the information efficiently and wrote the manuscript. YC deal with the information efficiently. CW, JS, and ZWa drew the original figures. LH, YS, and ZWi revised the manuscript. All the authors agree to be accountable for the content of the work.

## Conflict of Interest

The authors declare that the research was conducted in the absence of any commercial or financial relationships that could be construed as a potential conflict of interest.
